# Endoscopic Repair of Pharyngocutaneous Fistula Following Laryngectomy

**DOI:** 10.7759/cureus.5871

**Published:** 2019-10-09

**Authors:** Nikolay R Sapundzhiev, Lora T Nikiforova, Blagovesta H Spasova, Darina Ivanova, Boyan Balev

**Affiliations:** 1 Otolaryngology, Medical University of Varna, Varna, BGR; 2 Radiology, Medical University of Varna, Varna, BGR

**Keywords:** pharyngocutaneous fistula, laryngectomy, endoscopic repair

## Abstract

Pharyngocutaneous fistula (PCF) is a typical complication after total laryngectomy. It is managed predominantly via conservative techniques, but in cases of a large orifice or a substantial loss of surrounding soft tissue, surgical management is mandatory. Our aim was to apply a new endoscopic surgical approach for closure of a pharyngocutaneous fistula.

We report a case of a 61-year-old patient, who had been subjected to total laryngectomy with partial resection of tongue base and postoperative radiotherapy for advanced laryngeal carcinoma. Pharyngocutaneous fistula developed two years after the initial treatment. Barium swallow radiographs revealed a fistula between the neopharynx and the skin at the C2-C4 level. An endoscopic surgical repair was performed. Fat tissue harvested from the abdomen was injected into the area surrounding the pharyngeal opening of the fistula. The opening was sclerosed and sutured. The patient resumed a normal diet after several days and the fistula did not recur throughout the follow-up period.

The management of pharyngocutaneous fistula is mainly conservative and only in therapy-refractory cases, surgery is considered. In well-selected cases, an endoscopic approach can be used. Autologous fat injection around the hypopharyngeal opening of the PCF may be one of the possible options.

## Introduction

Pharyngocutaneous fistula (PCF) is a perceivable complication after total laryngectomy and represents an important cause for hospital morbidity, prolonged hospital stay, delayed initiation of postoperative radiotherapy and recurrent subsequent hospitalization [1]. The reported incidence is about 14.3% in primary laryngectomies and may reach 22.8% in patients with radiotherapy and salvage laryngectomies [2].

In the majority of the cases, closure of PCF can be achieved conservatively. However, if the PCF is large or a substantial loss of tissue is observed, surgical correction is required [3]. Conventional surgical techniques include reconstruction with highly vascular tissues such as regional flaps or free flaps [4]. Some authors have suggested endoscopic suturing of PCF as a less-invasive method [5].

This article reports a novel endoscopic surgical approach for the repair of PCF using autologous fat injection.

## Case presentation

A 61-year-old patient was referred for treatment to the Department of Neurosurgery and ENT diseases, St. Marina University Hospital, Varna, Bulgaria.

The patient had previously been subjected to laryngectomy at another institution with partial resection of the base of the tongue due to an advanced laryngeal carcinoma with infiltration of the hypopharynx. The postoperative period had been complicated with inflammation and fistulization. The fistula had been managed conservatively (without surgical intervention), but no precise information about the time of closure could be retrieved. A month after the surgery, the patient had undergone radiotherapy at a cumulative dose of 60 Gy. The oncologic follow-up had been uneventful. Two years later, chemotherapy (cisplatin, docetaxel) was attempted due to an intrathoracic relapse with compression of the trachea. During the treatment a pharyngocutaneous fistula developed, requiring a nasogastric tube to be placed. The patient presented to our department with dyspnea, hemoptysis, fistulization, intrathoracic compression of the trachea, and tracheostomy tube dysfunction.

**Figure 1:**
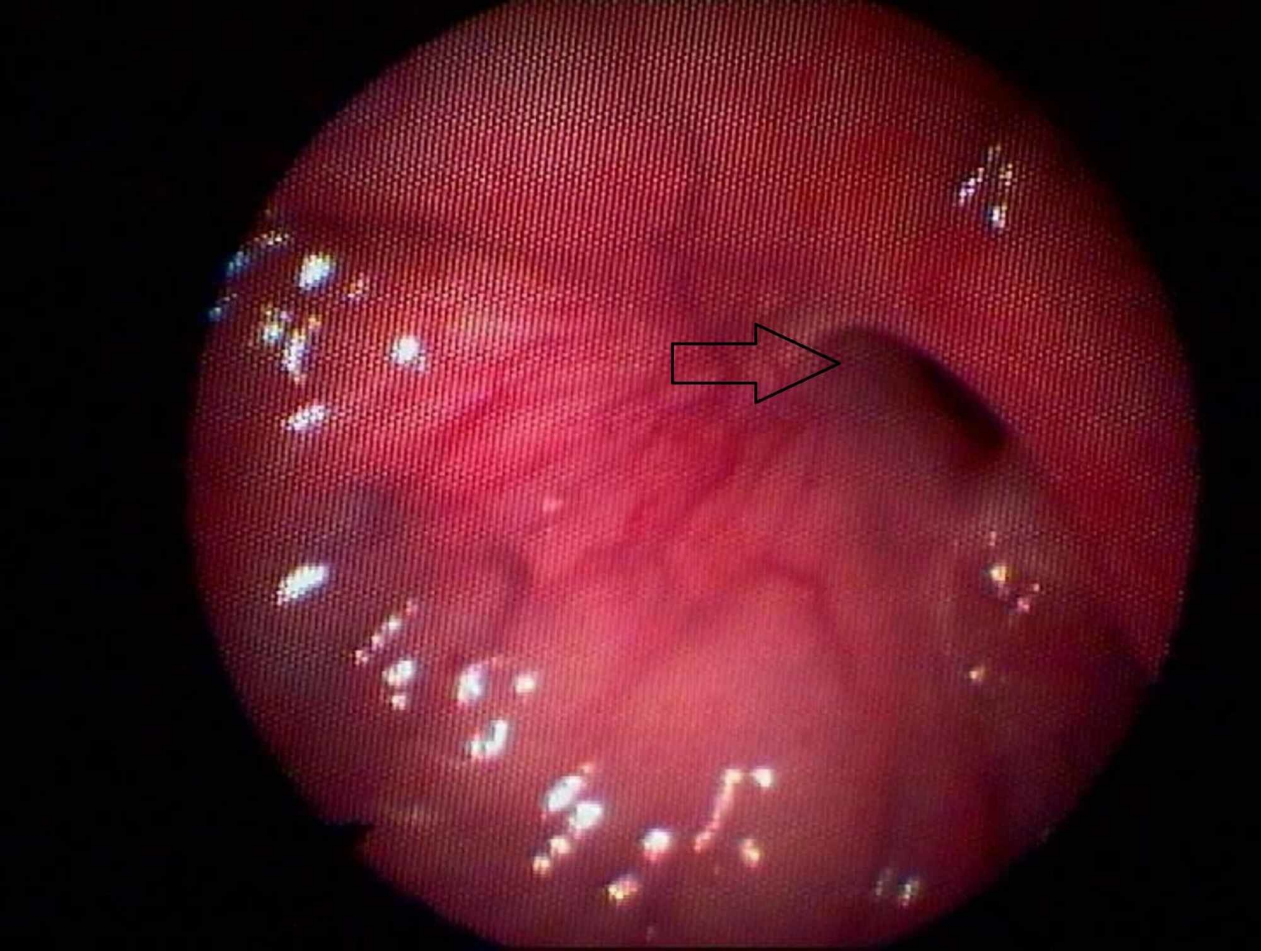
Rhinopharyngoscopy with a flexible scope The pharyngeal opening of the fistula is presented. The midline of the tongue is at the pointer of the scope (left bottom).

A flexible rhinopharyngoscopy revealed an epithelized opening, measuring approximately 4x4 mm at the junction between the base of the tongue and the hypopharynx (Figure 1).

**Figure 2:**
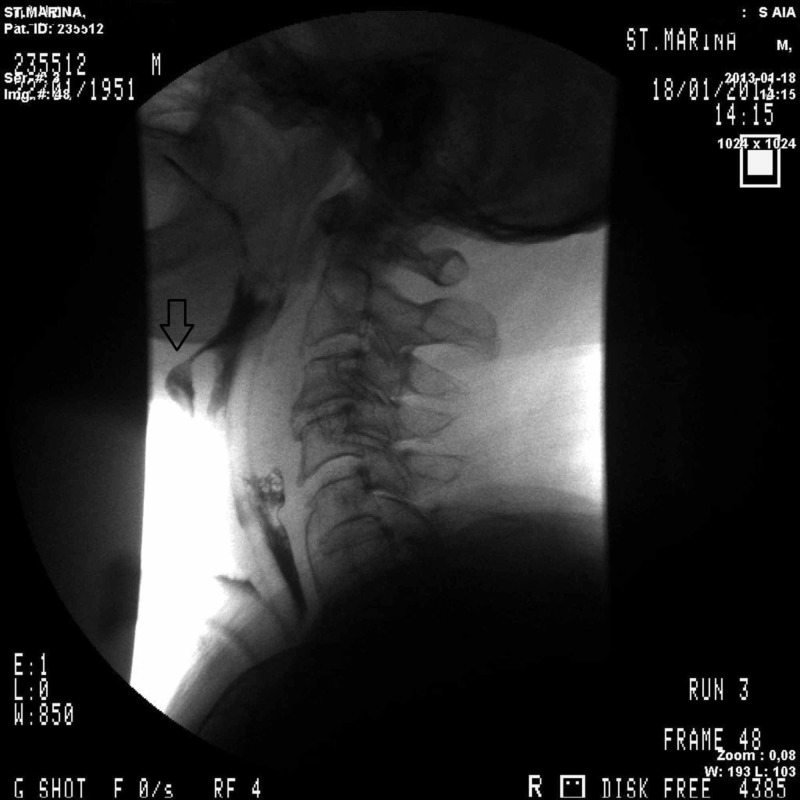
Barium swallow radiographs On sagittal view fistula between the neopharynx and the skin at the C2-C4 level is visualized. A nasogastric tube is in place.

Computed tomography (CT) scan showed no abnormalities in the base of the tongue and the hypopharynx. Biopsies of the PCF were negative. The patient was further evaluated with barium swallow radiography (Figure 2). 

The sagittal plane revealed a midline fistula between the pharynx and the skin at the level of C2-C4, with a narrow inlet at the junction between the tongue and the neopharynx, confirming the endoscopic finding.

**Figure 3:**
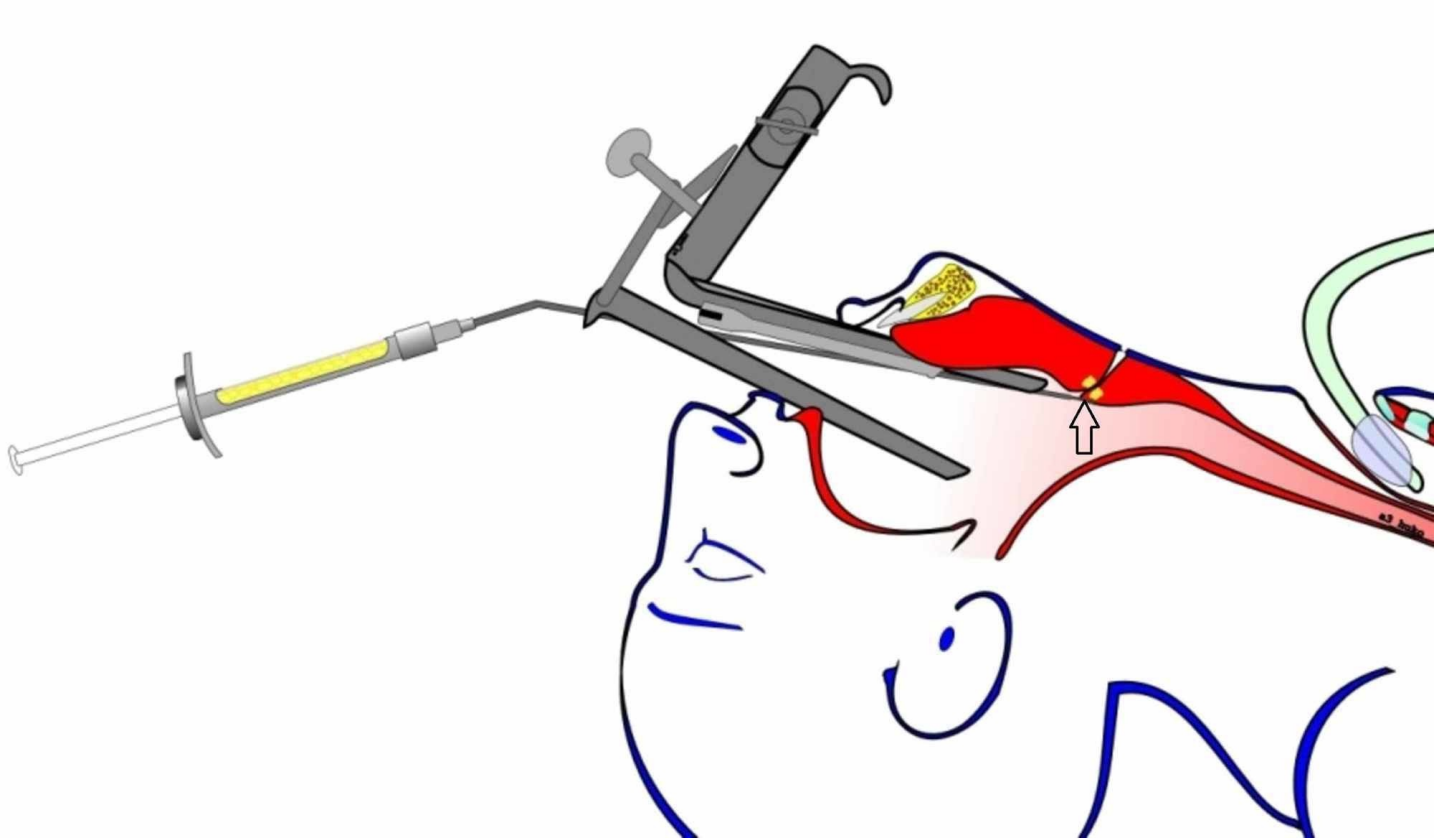
Endoscopic access and fat injection Endoscopic access to the neopharynx via Weerda distending laryngoscope. Autologous fat injection around the internal opening of the PCF.

An endoscopic surgical repair was attempted. The neopharynx was approached with the Weerda distending laryngoscope (Figure 3). 

Fat tissue harvested from the abdomen was processed by extracorporeal morcellation (minced into smaller pieces). Approximately 4 ml were injected into the area immediately surrounding the pharyngeal opening of the fistula using a Peretti angular injection cannula (Karl Storz GmbH & Co. KG, Tuttlingen, Germany). During the injection, the tissues felt soft, without marked induration after radiotherapy. Further, the internal opening was processed with electrocautery and sutured with a single Z-like fast-absorbing polyglactin.

The tracheal compression was managed with an extra-long cannula (Duravent XL, Andreas Fahl Medizintechnik-Vertrieb GmbH, Cologne, Germany). The patient resumed a normal diet four days after and the nasogastric tube was removed. For the next six months, the patient had normal oral feeding with no signs of leakage or inflammation around the area of the PCF.

Due to the intrathoracic progression of the disease leading to a fatal outcome, no subsequent follow-up was possible.

## Discussion

PCF is a frequent non-fatal, but a troublesome complication of total laryngectomy. It increases the treatment cost and morbidity rates, delays the adjuvant treatment and prolongs the hospital stay. It is also a risk factor for major neck vessel injury [6, 7].

In most cases, the fistula is treated with local wound care, compression and drainage. In 20-30% of cases conventional therapy is ineffective [3]. Such cases usually include large fistulas, substantial loss of surrounding soft tissue, insufficient pharyngeal mucosa or other causes leading to unsuccessful overall patient management, such as preoperative radiotherapy, prior tracheotomy or malnutrition [1,3,5,6].

Standard reconstruction techniques (regional or distant flaps) are characterized by high complication rates, frequent recurrent fistulizations and high morbidity [5]. Such complications may possibly be avoided with less aggressive surgical approaches in cases of comorbidities, advanced disease or ones not requiring large reconstructions [3,5].

In the first reported successful endoscopic closure of PCF, the neopharynx and the esophagus were accessed with a conventional intubation laryngoscope and further visualized with the aid of Hopkins Rod Telescopes and flexible esophagoscopes [5]. In the presented case, the pharyngeal fistula opening was accessed with the Weerda distending laryngoscope and a surgical microscope. This approach is well established and observed to be advantageous in terms of a larger visible field, space for surgical instruments and optimal retraction of the soft tissues [8]. The region of the intervention, which is difficult to access and manipulate, was above and in front of the tip of the diverticuloscope. It may be assumed that in patients with preserved superior teeth, limited mouth opening and other limiting anatomical conditions, the above approach may not suffice for adequate exposure of the PCF site.

In 2015, Fink et al. were the first to report an endoscopic approach for the treatment of pharyngocutaneous fistula [5]. Via multiple stitches quilting the fistula, and dental roll compressing skin/platysma to the pharynx, they achieved closure in five cases. Hespe et al. described a successful closure of PCF with the use of autologous fat graft injected into the area surrounding the external defect in one case [3]. The injection was performed percutaneously.

Fat grafting is a potential method for wound healing enhancement with unrevealed underlying mechanism [3]. Autologous fat transplantation has already been used for the rehabilitation of irritated tissues in the head and neck region, while an endoscopic reconstruction of PCF with autologous fat injection has not been previously reported [9]. In the reported patient, fat graft harvested from the abdomen was endoscopically injected in the soft tissues around the pharyngeal opening of the PCF, which was clearly identifiable and accessible with this approach. Subsequently, sclerosing and suturing of the opening was performed.

The major pathophysiological reason preventing these fistulas from healing by secondary intention is the saliva flushing the orifice. We deliberately focused on the internal opening of the fistula to try to stop the saliva penetrating the channel and thus allowing for the surrounding tissues to fuse and obliterate the fistula. Further, we sclerosed the opening with monopolar electrocautery. In an attempt to provide tight closure, a single suture was placed endoscopically. As the tissues were already altered (injected with fat) and the suture itself was loose, its role for the closure was deemed minimal. We assume that the transferred lipoaspirate initially compressed the opening of the fistula thus stopping the inflow of saliva and helping the sclerosing effect of the cautery. Additionally, the introduction of a population of adipose-derived stem cells may have reestablished a favorable wound-healing environment. The temporary interruption of chemotherapy may have also had a positive impact. The cisplatin and docetaxel-based regiment might have had a triggering role for this late apparition of the PCF. In this case, the repair strategy was successful - there was no leakage, inflammation or other complications and the patient returned to normal oral feeding. Later, after re-establishment of the chemotherapy, the PCF did not reoccur.

## Conclusions

In conservative therapy-refractory cases of pharyngocutaneous fistula, surgical intervention is mandatory. Conventional reconstruction methods are effective, but are connected with relatively high rate of complications and recurrent fistulizations. In well-selected cases, endoscopic repair may be attempted. Autologous fat injection around the hypopharyngeal opening of the PCF may be one of the possible methods, acting as a mechanical compression of the inlet of the fistula, as well as a promoter tissue for wound healing. However, more clinical experience in this field is required to judge the potential role of autologous fat grafting for the closure of persistent PCFs.
